# Effect of Heating Conditions during Moulding on Residual Stress–Strain Behaviour of a Composite Panel

**DOI:** 10.3390/polym14091660

**Published:** 2022-04-20

**Authors:** Andrii Kondratiev, Václav Píštěk, Oleksii Vambol, Pavel Kučera

**Affiliations:** 1 Department of Building Technology and Construction Materials, O.M. Beketov National University of Urban Economy in Kharkiv, Marshal Bazhanov Str. 17, 61002 Kharkiv, Ukraine; andrii.kondratiev@kname.edu.ua; 2 Institute of Automotive Engineering, Brno University of Technology, Technická 2896/2, 616 69 Brno, Czech Republic; pistek.v@fme.vutbr.cz; 3 Department of Composite Structures and Aviation Materials, National Aerospace University “Kharkiv Aviation Institute”, Chkalova Str. 17, 61070 Kharkiv, Ukraine; olexii.vambol@khai.edu

**Keywords:** process parameters, equipment, thermoelasticity, temperature differential

## Abstract

Currently, we observe extensive use of products made of polymeric composite materials in various industries. These materials are being increasingly used to manufacture large-sized structural parts that bear significant loads. However, increase in the volume of composites used in critical structures is impeded by the instability of properties of the resulting products. In most cases, the reason for this is the residual thermal stress–strain behaviour of the composite structure. This paper deals with the development of a method to predict the residual stress–strain behaviour depending on the heating conditions and distribution of the temperature field over the thickness of the moulded composite package. The method establishes the relationship between moulding process parameters and the effect of the auxiliary and basic equipment on the distribution of the temperature field, stresses, and strains in the moulded product. It is shown that the rate of temperature change at the stage of heating has its effect on the amount of residual deformation of the structure. Experimental studies have been carried out to determine the influence of several factors (rates of heating and cooling) on the residual deflection of the composite panel. Experimental data proves that specimens moulded under conditions of an increased heating rate get a greater deflection than those moulded at a lower heating rate. The error of results during the full-scale experiment did not exceed 6.8%. Our results provide an opportunity to determine the residual thermal stress–strain behaviour of the moulded structure with the required degree of accuracy without a series of experiments. It allows us to significantly simplify the practical implementation of the developed method and avoid any additional production costs.

## 1. Introduction

Recently, the share of structural elements made of polymeric composite materials (PCM) in products for various applications has significantly increased [1,2]. Introduction of PCM results in the reduction of the structure weight [3,4] and produces a product with the required specific properties (radio transparency, vibration resistance, low thermal conductivity, etc.) [5,6]. However, increase in the volume of PCM used in critical structures is impeded by the instability of properties of the resulting products (deviations in dimensions, structure, and properties of PCM) [7,8]. In most cases, the reason for this is the residual thermal stress–strain behaviour (SSB) of the composite structure [9].

As is known, the SSB of the structure made of PCM is formed during the technological process [10,11]. Occurrence of residual SSB is due to several factors, including the structure itself, number of layers, physic-mechanical characteristics (PhMC) of the moulded PCM package, monolayer thickness, etc. [12,13,14]. The above factors lead to an uneven distribution of the temperature field over the thickness of the product and, therefore, occurrence of residual SSB in the finished composite structure [15,16].

Recent studies refer to online monitoring of impregnation of the reinforcing material during the curing process [17,18,19]. The results allow us to model, and then to control and manage the curing process at the initial stage. However, occurrence of the thermal SSB at subsequent stages is not considered.

The rate of temperature change in the process of moulding of products of PCM in the process instructions is usually regulated at 2–3 °C/min [5,10]. However, these values cannot be used in the process in some cases [20]. First, this is due to occurrence of SSB in the moulded product, which can lead either to its failure [10,21,22], or to residual deformations exceeding the permissible ones [23,24]. 

In most cases, the results of recent studies represent a generalized solution pattern as, for example, in [11,25,26], or models which consider the individual factors only [27,28]. By way of example, the papers [27,28] describe the chemical and physical processes occurring in the moulded material in the curing process. Problems arising in the moulding process were considered, and the tasks were set, the completion of which allowed description of the chemical transformations in the binder, release of volatile products, and shrinkage processes. Nevertheless, impact of the temperature on the characteristics of the material during the curing process was not considered, and the influence of the product dimensions and geometry was not analysed. However, as a matter of practice, the proposed patterns simulate the moulding process in a general way, whereas the mathematical description of the ongoing physic-chemical processes is not commonly given or, at best, it is partially presented, which makes it difficult to implement them in practice in the production environment. Occurrence of relaxation and creep phenomena in the composite at the stage of temperature holding and curing are considered in a number of papers [29,30]. Distribution of the thermal field in the process of curing was not studied in these papers.

One of the primary issues related to modelling the curing process is being able to determine a model for changing the degree of curing, and hence the composite viscosity at the stage of its transition from a viscous-flow state to a solid one [31,32]. The papers [33,34] are of particular interest, since they give a description of SSB occurrence at the stage of PCM heating to the polymerization temperature, based on the experimental studies. However, these papers do not address the problems related to supply of additional heat to the composite, and nor the effect of the product geometry.

Temperature phenomena also participate in SSB at the stage of heating, along with shrinkage, as shown in [35]. Temperature stresses become comparable with shrinkage stresses upon reaching the material viscosity of 60–70% conversion in the binder.

A particular case of the composite curing by the autoclave method was studied in [36]. Detailed consideration of this method in the future would allow us to project the results onto other methods or to generalize.

It can be seen from the presented review that most papers did not consider the phenomenon of occurrence and effect of temperature stresses at the stage of heating of the material, and presence of reinforcing material in the product, and process parameters were determined by the PhMC of the binder only. This approach to determination of pro-cess parameters often leads to the violation of structure, appearance of unacceptable SSB in the material (product) and additional costs in the manufacturing of the PCM structures. In most cases, process parameters at the manufacturing site are determined by a trial-and-error method for any specific product. This is especially true for the stage of its development; therefore, the resulting process parameters are often not rational ones, in terms of production costs.

Manufacturing application of several techniques reduced the level of residual deformations in structures with an asymmetric design. However, during the process of moulding the PCM products with a symmetric structure, residual stresses and deformations of temperature origin occurred in some cases, and the product was not suitable for further use. In this regard, we need to model a moulding process that will describe the physic-chemical processes occurring in the moulded material, and that considers the factors affecting the residual SSB of the structure. Therefore, it is important to develop a method for determining the SSB depending on the heating conditions and distribution of the temperature field over the thickness of the moulded PCM package.

## 2. Materials and Methods

A thermosetting binder was investigated. This binder, after rejection, was treated as a solid body. A further increase in temperature did not lead to the transition of the matrix into an elastic, and a further increase did not lead to a viscous-flow state. When heated, the thermoset worked as a solid body up to the destruction temperature. The SSB of the PCM panel was studied based on the linear theory of thermoelasticity of an anisotropic body. To determine the SSB in the moulding process, the energy criterion was used. The following assumptions were made: the element was in the plane stress state, temperature effect was present, and there was no effect of the material shrinkage. The hypothesis of undeformed normals was accepted, and the hypothesis on absence of the pressure of composite panel layers on each other was considered true. The temperature field acting in the process of moulding onto the panel was assumed to be non-uniform over the thickness of the PCM package. Since the problem to be solved was the temperature problem, it was assumed that the total energy of the system was equal to the potential energy. Experimental studies were carried out in laboratory conditions using standard equipment, instruments, and fixtures (SNOL 60/300 NL Curing Oven, Utena, Lithuania). The influence of several factors (rates of heating and cooling) on the residual deflection of the PCM panel was studied experimentally on specimens of plates of 150 × 150 mm. The specimens were made of prepreg based on T-10-14 glass cloth (Producer: JSC “Polotsk-Steklovolokno”, Polotsk, Republic of Belarus) on FP-520 binder (Producer: Federal State Unitary Enterprise All-Russian Scientific Research Institute of Aviation Material, Russian Federation) by manual layup onto a flat mould. The moulded PCM package was a symmetrical structure [0°; 90°; 90°; 0°]. Deflections were measured by photographs. The specimen was fixed before the lens in the way that the curved plate located in front of the lens was projected into a clear curve.

## 3. Theoretical Background

**Figure 1:**
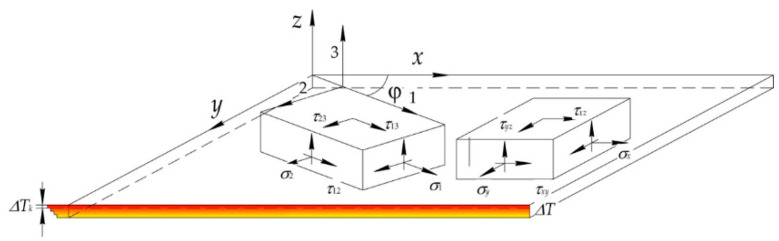
Element of unidirectional layer of PCM in axes 1, 2, 3 related to the direction of reinforcement, and axes *x*, *y*, *z*.

The main sources of residual stress for PCM structures in the process of moulding are the differences in the thermophysical and elastic properties of the components of the material. Based on the principle of maintaining the integrity of the PCM structure during the process of deformation when moulding on the contact surfaces the layers for displacements (*u_x_*, *u_y_*, *u_z_*) and tangential stresses (*τ_xz_*, *τ_yz_*), the following relations should be fulfilled (Figure 1) [37,38]:

(1) $$u_{x}^{\left(i+1\right)}=u_{x}^{\left(i\right)};\;u_{y}^{\left(i+1\right)}=u_{y}^{\left(i\right)};\;u_{z}^{\left(i+1\right)}=u_{z}^{\left(i\right)};\;\tau_{xz}^{\left(i+1\right)}=\tau_{xz}^{\left(i\right)};\;\tau_{yz}^{\left(i+1\right)}=\tau_{yz}^{\left(i\right)}.$$

Further, we accepted the hypothesis of undeformed normals represented as follows [37,39]: *ε_z_* = 0; *γ_xz_* = 0; *γ_yz_* = 0. In addition, we considered the hypothesis of no pressure of the layers of the composite panel on each other to be true, i.e., *σ_z_* = 0.

Based on the assumptions made, physical relationships for the layer reinforced at the angle *φ* to axis *Ox*, are written as:

(2) $${\left[\begin{array}{c} \sigma_{x}\\ \sigma_{y}\\ \tau_{xy} \end{array}\right]}_{k}={\left[\begin{array}{ccc} b_{11} & b_{12} & b_{13}\\ b_{21} & b_{22} & b_{21}\\ b_{31} & b_{32} & b_{33} \end{array}\right]}_{k}\cdot \left[\begin{array}{c} \varepsilon_{x}\\ \varepsilon_{y}\\ \gamma_{xy} \end{array}\right]-{\left[\begin{array}{c} a_{T1}\\ a_{T2}\\ a_{T3} \end{array}\right]}_{k}{\left[\Delta T\right]}_{k},$$

where *b_ij_*, *a_ij_*—constants defined as [38,39]; $\Delta T_{i}$—temperature differential in the *i*–th layer.

Temperature differential across the thickness of PCM package depends on the temperature characteristics of the shaping surface and auxiliary equipment as follows [40,41]:

(3) $$\Delta T=\frac{\left[\left(\frac{\delta_{1}}{\lambda_{1}}+\frac{1}{\alpha_{1}}\right)-\left(\frac{\delta_{3}}{\lambda_{3}}+\frac{1}{\alpha_{3}}\right)\right]\frac{\delta_{2}}{\lambda_{2}}\nu_{p}}{2\left(\frac{1}{\alpha_{1}}+\frac{1}{\alpha_{3}}+\frac{\delta_{1}}{\lambda_{1}}+\frac{\delta_{2}}{\lambda_{2}}+\frac{\delta_{3}}{\lambda_{3}}\right)}\left[\frac{\left(\frac{c_{3}\rho_{3}\delta_{3}^{2}}{\lambda_{3}}+\frac{2c_{3}\rho_{3}\delta_{3}}{\alpha_{3}}\right)-\left(\frac{c_{1}\rho_{1}\delta_{1}^{2}}{\lambda_{1}}+\frac{2c_{1}\rho_{1}\delta_{1}}{\alpha_{1}}\right)}{\left(\frac{\delta_{1}}{\lambda_{1}}+\frac{1}{\alpha_{1}}\right)-\left(\frac{\delta_{3}}{\lambda_{3}}+\frac{1}{\alpha_{3}}\right)}-c_{2}\rho_{2}\delta_{2}\right],$$

where $\delta_{1},\;\alpha_{1},\;\lambda_{1},\;c_{1},\rho_{1},\delta_{2},\;\lambda_{2},\;c_{2},\;\rho_{2},\;\delta_{3},\;\alpha_{3},\;\lambda_{3},\;c_{3},\rho_{3}$—thickness, heat transfer and thermal conductivity coefficients, specific heat capacity, density of the shaping surface, PCM package and auxiliary equipment, respectively; *ν_p_*—heating rate.

In some cases, a double heat inlet, as in the autoclave or oven, cannot be implemented, for example, repairs of structures where the heating blanket acts as a heating medium, or in the case of moulding with the heated equipment [41,42]. In this case, the PCM package is heated on one side only, and the dependence Equation (3) is simplified:

(4) $$\Delta T=\frac{c_{2}\rho_{2}\delta_{2}^{2}}{k\lambda_{2}}\nu_{p},$$

where *k* = 2 (in case of one-sided heating of the material).

Deformations arising in the panel are defined as follows:

(5) $$\varepsilon_{x}=\varepsilon_{x}^{0}+\chi_{x}z;\;\varepsilon_{y}=\varepsilon_{y}^{0}+\chi_{y}z;\;\gamma_{xy}=\gamma_{xy}^{0}+\chi_{xy}z,$$

where $\varepsilon_{x}^{0},\;\varepsilon_{y\;,}^{0}\;\gamma_{xy}^{0}$—deformations in the median plane;$\chi_{x}=-\frac{\partial^{2}w}{\partial x^{2}};\;\chi_{y}=-\frac{\partial^{2}w}{\partial y^{2}};\;\chi_{xy}=-2\frac{\partial^{2}w}{\partial x\partial y}$—curvatures.

**Figure 2:**
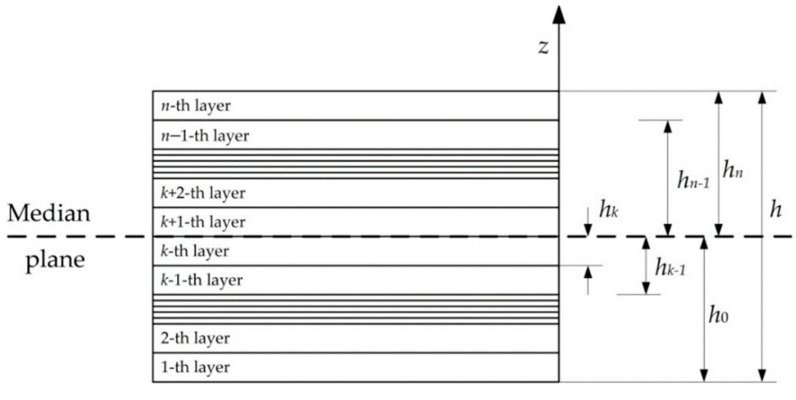
Coordinates of layers.

Deformation energy of the panel is written as [34]:

(6) $$U=\iint_{S}\int_{0}^{h}\left(\sigma_{x}\varepsilon_{x}+\sigma_{y}\varepsilon_{y}+\tau_{xy}\gamma_{xy}\right)dzdS\;,$$

where $\sigma_{x},\;\sigma_{y},\;\tau_{xy}$—internal stresses in the panel determined from dependencies Equation (2); $\varepsilon_{x},\;\varepsilon_{y},\gamma_{xy}$—deformations of the panel determined from dependencies Equation (5); *h*—thickness and *S*—area of the panel; $\frac{h_{k}+h_{k-1}}{2}={\bar{z}}_{k}$—distance from median surface of the package to the middle of *k*–th layer (Figure 2).

Substituting Equation (5) in Equation (6), we obtain the expression below:

(7) $$U=\iint_{S}\left(N_{x}\varepsilon_{x}^{0}+N_{y}\varepsilon_{y}^{0}+N_{xy}\gamma_{xy}^{0}+M_{x}\chi_{x}+M_{y}\chi_{y}+M_{xy}\chi_{xy}\right)dS,$$

where $N_{x},\;N_{y},\;N_{xy},\;M_{x},\;M_{y},\;M_{xy}$—forces and moments acting within the panel [38,39].

The resulting forces and moments acting in the system are defined as follows [38,39]:

(8) $$\left[\begin{array}{c} N_{x}\\ N_{y}\\ N_{xy}\\ M_{x}\\ M_{y}\\ M_{xy} \end{array}\right]=\left[\begin{array}{ccccccc} B_{11} & B_{12} & B_{13} & & C_{11} & C_{12} & C_{13}\\ B_{21} & B_{22} & B_{23} & & C_{21} & C_{22} & C_{23}\\ B_{31} & B_{32} & B_{33} & & C_{31} & C_{32} & C_{33}\\ C_{11} & C_{12} & C_{13} & & D_{11} & D_{12} & D_{13}\\ C_{21} & C_{22} & C_{23} & & D_{21} & D_{22} & D_{23}\\ C_{31} & C_{32} & C_{33} & & D_{31} & D_{32} & D_{33} \end{array}\right]\cdot \left[\begin{array}{c} \varepsilon_{x}^{0}\\ \varepsilon_{y}^{0}\\ \gamma_{xy}^{0}\\ \chi_{x}\\ \chi_{y}\\ \chi_{xy} \end{array}\right]-\left[\begin{array}{c} B_{T1}\\ B_{T2}\\ B_{T3}\\ D_{T1}\\ D_{T2}\\ D_{T3} \end{array}\right],$$

where $B_{ij}$, $C_{ij},\;D_{ij}$,$\;B_{Ti}$, $D_{Ti}$—constants defined as [38,39]:

After substitution of Equation (8) in Equation (7) we obtain the following dependence:

(9) $$U=f\left(\varepsilon_{x}^{0},\;\varepsilon_{y}^{0},\gamma_{xy}^{0},\chi_{x},\;\chi_{y},\chi_{xy},B_{ij},C_{ij},\;D_{ij},B_{Ti},D_{Ti}\right),$$

Deflection function is represented as the power series below:

(10) $$w=\left(x^{2}+y^{2}\right)\left(f_{1}+f_{2}x^{2}+f_{3}y^{2}\right),$$

where $f_{1},\;f_{2},\;f_{3}—$some of coefficients.

The temperature field acting in the process of moulding onto the panel is assumed to be non-uniform over the thickness of the PCM package. Since the problem being solved is the temperature problem, it makes no sense to look for the work of external forces, which means that the total energy of the system will be equal to the potential energy. Substituting the deflection Equation (10) into Equation (5), and then into energy Equation (9), we obtain, after integration of the latter, the dependence of deformation energy on the unknown coefficients and deformations as:

(11) $$U=U\left(f_{1},f_{2},\;f_{3},\;\varepsilon_{x}^{0},\;\varepsilon_{y}^{0},\gamma_{xy}^{0}\right).$$

Dependence Equation (11) is functional, and its minimum will be reached when all derivatives of the total energy regarding coefficients of series Equation (10) are set to zero. Then the re-solving system of equations will take the form:

(12) $$\frac{\partial U}{\partial f_{1}}=0;\;\;\frac{\partial U}{\partial f_{2}}=0;\;\;\frac{\partial U}{\partial f_{3}}=0;\;\frac{\partial U}{\partial \varepsilon_{x}^{0}}=0;\;\;\frac{\partial U}{\partial \varepsilon_{y}^{0}}=0;\;\;\frac{\partial U}{\partial \gamma_{xy}^{0}}=0.$$

This system represents the linear algebraic equations; the number of these equations will always be equal to the number of coefficients. The obtained coefficients are substituted into Equation (10), and then into Equations (2) and (5), which is the full solution to the problem.

**Figure 3:**
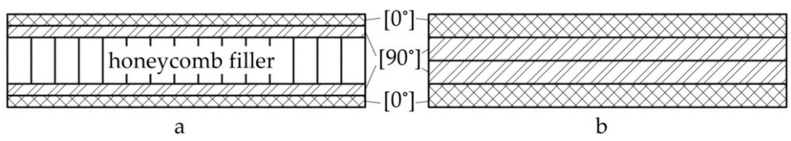
Layup pattern for sandwich panel (**a**) and laminated panel (**b**).

As an example, we determined the deflection and stresses in a PCM panel of 100 × 100 mm with a symmetrical layup [0°; 90°]. Three options for the panel were considered (Figure 3):

- sandwich panel with honeycomb filler of 4 mm high and four bearing layers of glass cloth (Figure 3a);
- sandwich panel with honeycomb filler of 9 mm high and four bearing layers of glass cloth (Figure 3a);
- glass cloth laminated panel with a monolayer of 0.25 mm thickness (Figure 3b).

The following PhMC of the T-10-14 glass cloth on FP-520 binder were taken in the calculation: *E*_1_ = 30.8 GPa; *E*_2_ = 25.5 GPa; *G*_12_ = 3.1 GPa; *μ*_12_ = 0.28; *ρ* = 1700 kg/m^3^; *α*_1_ = 4 × 10^−6^ K^−1^; *α*_2_ = 6 × 10^−6^ K^−1^; *c* = 850 J/(kg·K); *λ* = 0.3 W/(m·K); *T* = 150–160 °C; *δ* = 0.21 mm [43].

**Figure 4:**
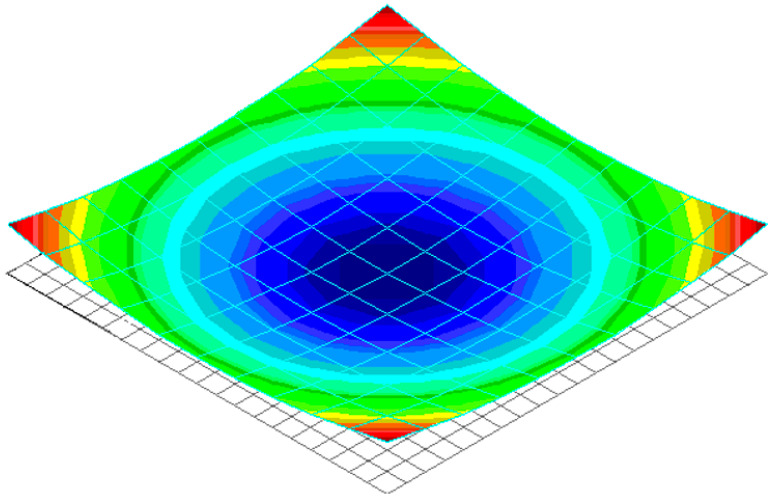
General view of deflection of the panel with symmetric reinforcement structure because of uneven heating across the thickness.

For the presented material, reinforcement angles, and the nature of fastening, the surface of the panel, will take the form shown in Figure 4, due to uneven heating across the thickness.

Results of the calculation are shown in Table 1, Table 2 and Table 3.

According to the resulting data, maximum values of deflections both for the laminated and sandwich panels were observed at the corners of the plate. 

For the sandwich panel in the three various heating modes, there was no deflection. This can be explained by the high rigidity of the sandwich panels. However, with the increase in the rate of temperature change, such panels featured higher stresses compared to the laminated structures. With the increase in the thickness of the sandwich panel and the higher rate of temperature change, these stresses became critical and led to the failure of the adhesive joint between the honeycomb filler and bearing layers [8] or caused delamination in the bearing layers of the panels [44].

## 4. Experimental Research

Experimental studies have been carried out regarding the effect of rates of heating and cooling on the residual deflection of the PCM panel. 150 × 150 mm plates were taken as specimens. The specimens were made of prepreg based on T-10-14 glass cloth on FP-520 binder by manual layup on a flat mould, with a symmetric structure for the moulded package [0°; 90°; 90°; 0°].

**Figure 5:**
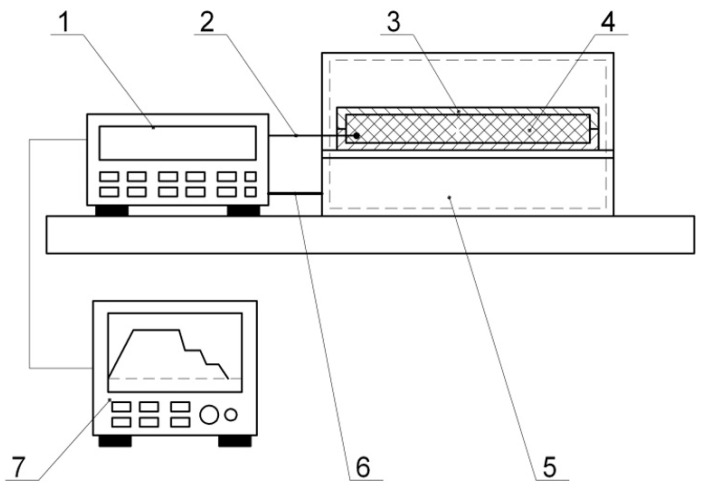
Test bench layout: 1—thermostat (TERMOTET 04/2); 2—thermocouple; 3—shaping surface; 4—moulded PCM package; 5—SNOL 60/300 NL Curing Oven; 6—supply; 7—data recorder (computer with the special soft).

Moulding was carried out on a flat fixture of a 10 mm thick, polished steel plate. The degreased surface of the fixture was coated with an antiadhesive layer of lubricant, on which the layers of the moulded package were successively laid according to the reinforcement pattern. The layers, arranged as above, were pressed with a roller. A vacuum cover, fixed along the perimeter with a putty yarn, was placed on the laid PCM package. The prepared package was placed under vacuum and put into the heater (Figure 5).

Moulding was carried out on a flat fixture of a 10 mm thick, polished steel plate. The degreased surface of the fixture was coated with an antiadhesive layer of lubricant, on which the layers of the moulded package were successively laid according to the reinforcement pattern. The layers, arranged as above, were pressed with a roller. A vacuum cover, fixed along the perimeter with a putty yarn, was placed on the laid PCM package. The prepared package was placed under vacuum and put into the heater (Figure 5).

On completion of the moulding process, an external examination of specimens was carried out to record visible defects, after which the specimen deflection was measured using the obtained photographs. The specimen was fixed before the lens such that the curved plate located in front of the lens was projected into a clear curve.

**Figure 6:**
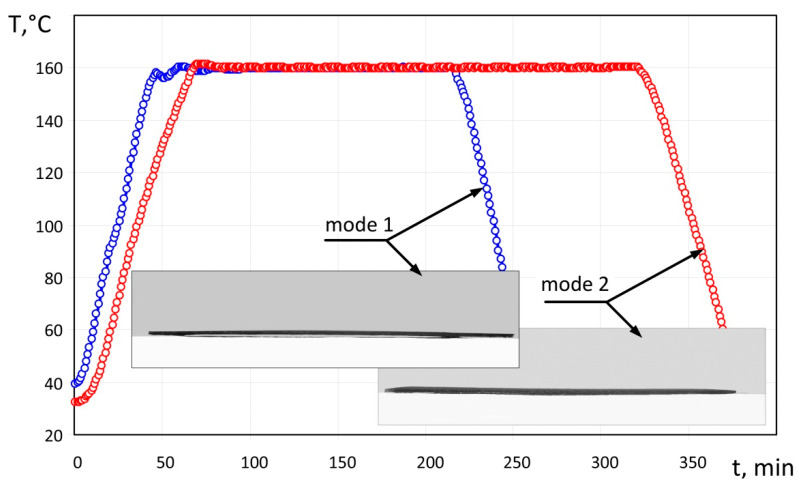
Moulding modes No. 1 and No. 2, and specimens obtained with the use of these modes.

The specimens obtained according to moulding mode No. 1 were made according to the vacuum moulding conditions as shown on the graph (Figure 6), at a heating rate of 2.8 °C/min and a cooling rate of 3.5 °C/min. On completion of the moulding mode, the specimen (Figure 6) had a smooth surface and dense structure, with no visible defects (delamination of edges, waviness, or swelling); deflection of the resulting specimen was equal to 1.9 mm.

The specimens obtained according to moulding mode No. 2 were made as shown on the graph in Figure 6, at a heating rate of 2 °C/min and a cooling rate of 2.3 °C/min. On completion of the moulding mode, the specimen (Figure 6) had a smooth surface and dense structure, with no visible defects (delamination of edges, waviness, or swelling); deflection of the resulting specimen was equal to 0.7 mm.

**Figure 7:**
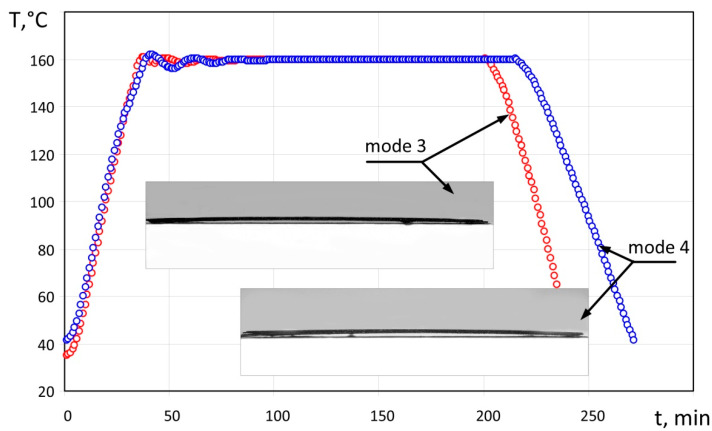
Moulding modes No. 3 and No. 4, and specimens obtained with the use of these modes.

**Figure 8:**
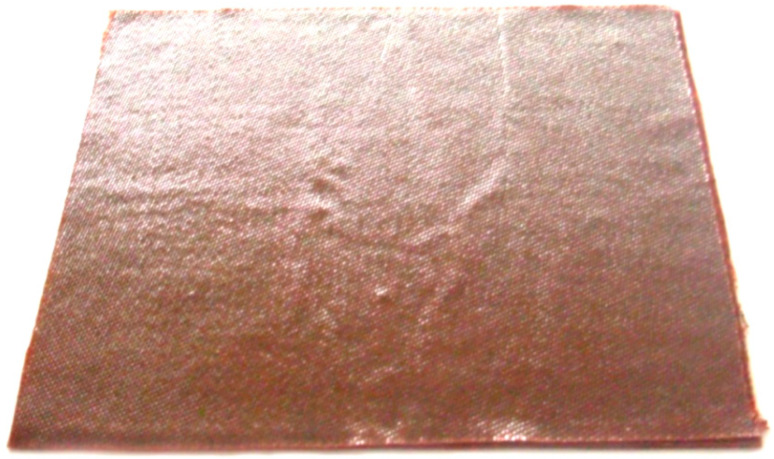
Upper surface of the specimen obtained with the use of moulding mode No 3.

The specimen obtained according to moulding mode No 3 was made as shown on the graph in Figure 7, at the heating rate of 3.6 °C/min and cooling rate of 3.5 °C/min. On completion of the moulding mode, the specimen had several visible defects, namely, delamination of edges, surface waviness and swelling areas (Figure 8). In the transverse direction we observed a zone with noticeable delamination of the upper layer from the lower one passing through the central section. The specimen deflection was equal to 2.3 mm.

The specimen obtained according to moulding mode No. 4 was made as shown on the graph in Figure 7, at a heating rate of 3.8 °C/min and a cooling rate of 2.5 °C/min. On completion of the moulding mode, the specimen had a smooth surface without swelling; however, slight delamination was observed along its edges; the deflection of the resulting specimen was equal to 2.5 mm.

The results of the experiment are presented in Table 4.

Summarizing the experimental results, we can say that with an increase in the heating rate from 2.0 to 3.8 °C/min (specimens obtained with the use of moulding modes No. 2, 1, 3, 4) the deflection increased from 0.7 to 2.5 mm. This is primarily due to stresses occurring in the heating stage, which caused the irreversible change in the panel shape. 

Cooling at a higher rate led to violations in the integrity, continuity and several defects in the material (product), especially if the previous stage of the moulding process (heating stage) was carried out at a higher rate. Specimens obtained with the use of moulding modes No. 3 and No. 4 were made in the conditions of increased heating rate. For example, whereas the specimen obtained according to moulding mode No. 4 and cooled at 2.5 °C/min showed insignificant delamination of the edges, the specimen obtained with the use of moulding mode No. 3 and cooled at 3.5 °C/min, showed more severe delamination of edges, surface waviness and poor adhesion of the upper layer. Even though the amount of deflection of the No. 4 specimen was somewhat larger than that of the No. 3 specimen, a higher rate of cooling of the No. 3 specimen led to delamination of its surface. Regarding the moulded structure, high cooling rates did not lead to the occurrence of residual deflections (for symmetric structures) but caused the structure failure. 

During these studies, we also obtained the dependence of panel deflection on the rate of temperature change (Table 5). Panel deflection was determined depending on the rate of temperature change for the range from 20 to 200 °C.

As can be seen from the results, the amount of panel deflection increased with the increase in the heating rate. However, with the temperature rise there was a slight increase in the amount of deflection. First, this is due to the influence of temperature on the properties of the moulded material. As is known [10,41], the elastic and strength characteristics of the material in the transverse direction become lower with an increase in temperature, while the thermal characteristics increase. In our case, growth of the values of the thermal characteristics of the material prevailed over the decrease in the elastic ones in the temperature range of 20–100 °C. As a result, deflection of the panel in the mentioned temperature range increased. However, with the further temperature rise, the decrease in elastic characteristics was more intense than the growth of thermal ones. Therefore, stiffness of the material became lower, while its compliance increased, which led to a decrease in deflection. It can be said that when the residual SSB is determined at the stage of designing a structure, and characteristics of the material are assumed constant in the operating temperature range, the value of the technological deflection (in our case) will be somewhat overestimated.

## 5. Results and Discussion

Comparing the theoretical and experimental results (Table 6), we can say the following. The error between the theoretical values of deflections obtained at the temperature of 20 °C ($w_{t}^{20{}^{\circ}C}$) and their experimental values (*w_e_*) for specimens obtained according to mode No. 2–4 were overestimated by 17–18%, and for specimen No. 1 the deflection turned out to be overestimated by 5%. The overestimation of the theoretical values of the deflection sizes is mainly due to the effect of temperature on the PhMC materials; as well as creep phenomena and relaxation processes not being considered when determining the deflections.

When the deflections were determined, considering the change in the PhMC of the material (product) depending on temperature ($w_{t}^{20{}^{\circ}C}$), the error between the theoretical and experimental results for specimens No. 1–4 decreased and amounted to a maximum of 77%. 

**Figure 9:**
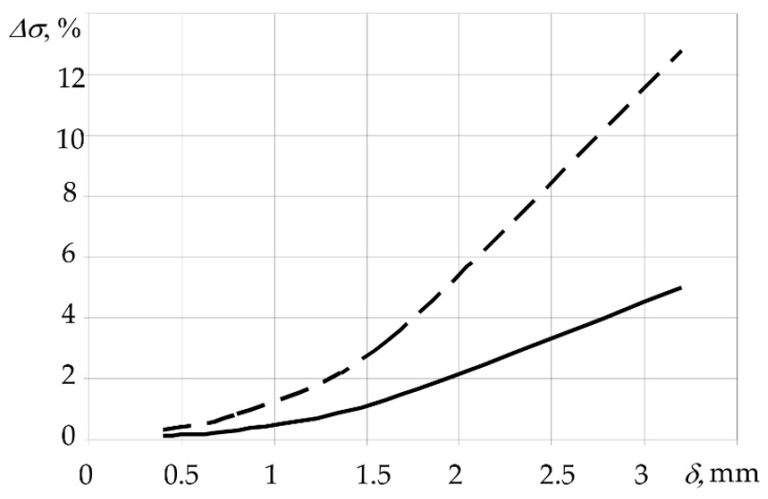
Change in maximum stresses calculated considering the rate of temperature change (*ν_p_*) and thickness of moulded package: ––– *ν_p_* = 2 °C/min; – – *ν_p_* = 5 °C/min.

Thus, the rate of temperature change should be chosen for each specific case based on the materials used, dimensions of the structure, and the PCM reinforcement pattern. In addition, the choice of process parameters depends on the shaping surface, auxiliary equipment, and heating medium. It should be noted that uneven distribution of temperature over the thickness of the panel has practically no effect on the amount of deflection during moulding of thick-walled structures or structures of high rigidity, since stresses are the determining parameter in this case. Accuracy of stress calculation depending on thickness of the moulded material for the rates of temperature rise is shown in Figure 9.

To obtain thin-walled structures with high accuracy or contours, it is unacceptable to neglect the unevenness of the temperature field across the thickness because of deflections, which occur after removal of the product from the shaping surface. For the laminated structures under study, deflections at the rate of temperature change of more than 1–2 °C/min were not acceptable, since the allowable deflection should not exceed 1–2 mm, and for high-precision dimensionally stable structures the allowable deflection should be a maximum of 0.1 mm [2,34].

## 6. Conclusions and Further Research

A new method for determining the SSB depending on heating conditions and the distribution of the temperature field over the thickness of a moulded PCM package has been developed. The method establishes the relationship between moulding process parameters and the effect of the auxiliary equipment and shaping surface on the distribution of the temperature field and the SSB in the moulded product.

The rate of temperature change at the stage of heating has its effect on the amount of residual deformation of the structure. As shown by the experimental data, specimens moulded under conditions of an increased heating rate featured higher deflection than those moulded at a lower heating rate.

The error of results obtained during the numerical experiment based on the models and results of the full-scale experiment did not exceed 6.8%.

Our results enable the calculation of the residual SSB of a moulded structure with the required degree of accuracy without a series of experiments and, accordingly, significantly simplify the practical implementation of the developed method and avoid any additional production costs.

The proposed calculation pattern describes the SSB occurring in smooth panel structures, however, replacing the mathematical model for calculating the plate by another pattern for a beam, rod, or skin, will allow us, in the future, to calculate the SSB in the moulding process, considering the uneven distribution of temperature over the product thickness. It is necessary to note that calculations of the residual SSB in asymmetric structures should consider the difference between the initial and final temperatures of the moulding process, in addition to the temperature differential across the thickness of the PCM package.

## Figures and Tables

**Table 1:** Dependence of maximum deflection (mm) of the panel on the heating rate.

Heating Rate, °C/min	PCM Package Thickness, mm		
	Sandwich Panel		Laminated Panel
	5	10	1
3	0.04	0.019	3.2
2	0.03	0.013	2.1
1	0.01	~0.00	0.98

**Table 2:** Dependence of maximum stresses (MPa) in the layers of sandwich panel on the heating rate.

Heating Rate, °C/min	Layers							
	Panel of 5 mm Thick				Panel of 10 mm Thick			
	No 1	No 2	No 3	No 4	No 1	No 2	No 3	No 4
3	2.9	1	0.03	−0.1	13.9	4.3	−0.05	−0.52
2	1.9	0.6	0.02	−0.07	8.8	2.9	−0.04	−0.35
1	0.95	0.31	0.01	−0.04	4.7	1.5	−0.02	−0.1

**Table 3:** Dependence of maximum stresses (MPa) in the layers of panel on the heating rate.

Heating Rate, °C/min	Layers			
	No 1	No 2	No 3	No 4
3	0.32	0.08	0.05	−0.015
2	0.21	0.06	0.03	−0.01
1	0.11	0.03	0.02	−0.005

**Table 4:** Processing of results of the experiment.

Moulding Mode	Heating Rate, °C/min	Cooling Rate, °C/min	Deflection, mm	Notes
No 1 (Figure 6)	2.8	3.5	1.9	Surface is smooth, without any visible defects.
No 2 (Figure 6)	2.0	2.3	0.7	Surface is smooth, without any visible defects.
No 3 (Figure 7 and Figure 8)	3.6	3.5	2.3	Surface is wavy with swellings. Delamination of edges and partial delamination of the upper layer is observed.
No 4 (Figure 7)	3.8	2.5	2.5	Surface is smooth, without any visible defects. Slight delamination is observed on edges of the specimen.

**Table 5:** Dependence of deflection of the panel (150 × 150 mm) on the rate of temperature change, mm.

Heating Rate, °C/min	Temperature, T, °C				
	20	50	100	150	200
1	0.21	0.23	0.23	0.19	0.13
2	0.83	0.89	0.89	0.75	0.5
3	2.14	2.30	2.30	1.95	1.30
4	3.11	3.35	3.34	2.83	1.89

**Table 6:** Theoretical and experimental data processing.

**Moulding Mode**	$w_{e}$ **, mm**	$w_{t}^{20{}^{\circ}C},\;\mathrm{mm}$	$w_{t}^{160{}^{\circ}C},\;\mathrm{mm}$	$\frac{w_{t}^{20{}^{\circ}C}-w_{e}}{w_{e}}100,\%$	$\frac{w_{t}^{160{}^{\circ}C}-w_{e}}{w_{e}}100,\;\%$
1	1.9	2.00	1.82	5.3	4.2
2	0.7	0.83	0.75	18.6	7.1
3	2.3	2.70	2.45	17.4	6.5
4	2.5	2.93	2.67	17.2	6.8

## Data Availability

Not applicable.
